# Ductus venosus reversed flow in omphalocele: Could it be a prognostic factor for long-term neurological impairment?

**DOI:** 10.25122/jml-2021-0344

**Published:** 2021

**Authors:** Roxana Elena Bohîlțea, Nicolae Bacalbașa, Bianca Margareta Mihai, Corina Grigoriu, Consuela-Mădălina Gheorghe, Tiberiu Augustin Georgescu, Irina Maria Vlădăreanu, Valentin Varlas

**Affiliations:** 1. Department of Obstetrics and Gynecology, Carol Davila University of Medicine and Pharmacy, Bucharest, Romania; 2. Department of Obstetrics and Gynecology, Filantropia Clinical Hospital, Bucharest, Romania; 3. Department of Obstetrics and Gynecology, University Emergency Hospital Bucharest, Bucharest, Romania; 4. Department of Marketing and Medical Technology, Carol Davila University of Medicine and Pharmacy, Bucharest, Romania; 5. Department of Pathology, Carol Davila University of Medicine and Pharmacy, Bucharest, Romania; 6. Faculty of Medicine, Carol Davila University of Medicine and Pharmacy, Bucharest, Romania

**Keywords:** omphalocele, congenital abdominal wall defects, ductus venosus reversed flow, neurological impairment, OEIS complex – omphalocele, exstrophy of the bladder, imperforate anus, spinal defects complex, AFI – amniotic fluid index, AC – abdominal circumference, C-section – Cesarean section

## Abstract

Omphalocele (exomphalos) represents one of the most frequent congenital abdominal wall defects. It presents as a defect of inconstant size and is located on the midline, at the base of the umbilical cord, the skin, fascia, and abdominal muscles being absent at this level. Omphaloceles are classified as liver-containing or non-liver-containing, the latter containing primarily bowel loops. We present the case of a 37-year-old pregnant woman with an early diagnosis of liver-containing omphalocele associating ductus venosus reversed flow, with the aim to highlight the importance of the first-trimester morphology scan and to develop a pilot study regarding the neurological development of infants after surgical repair of giant omphaloceles. The particularity of this case consists of a fetus with a positive diagnosis of a giant liver-containing omphalocele but with a small abdominal wall defect during the first-trimester morphology scan at 13 weeks and 3 days of gestation which associated ductus venosus reversed flow, presenting a normal karyotype postabortum. With a small defect, we can speculate the risk of strangling besides the mechanical traction exercised on the ductus venosus generating fetal distress, specifically fetal hypoxia at an early gestational age. In conclusion, the main issue, in this case, was if the fetal omphalocele and ductus venosus reversed flow indicated fetal hypoxia, what was the obstruction effect on the oxygenated blood pathway caused by the abdominal defect, and which were the long-term effects on infants with this complex pathology with an unknown outcome.

## Introduction

Omphalocele (exomphalos) represents one of the most frequent congenital abdominal wall defects. It presents as a defect of inconstant size and is located on the midline, at the base of the umbilical cord, the skin, fascia, and abdominal muscles being absent at this level [1]. Omphaloceles are covered by the amnion, Wharton’s jelly, and peritoneum. The apex of the sac presents the umbilical cord insertion; the sac usually contains herniated abdominal organs: midgut, liver, spleen, or even gonads [2]. Omphaloceles are classified as liver-containing or non-liver-containing, the latter containing primarily bowel loops. The size of the omphalocele is between 2–10 cm, and 40–80% of cases have a minimum of one associated anomaly [3]. Furthermore, giant omphaloceles are described as omphaloceles that contain more than 75% of the liver or a defect size more than 5 cm [3].

Omphaloceles have an incidence of 2 per 10,000 live births in the United States and 2.6 per 10,000 births worldwide [4]. Women at the extreme reproductive ages (<20 years old and >40 years old) have a twofold higher risk of delivering a newborn with omphalocele than the general reproductive population [5, 6], and women of color present a higher prevalence than white women (1.91 *vs.* 1.47 per 10,000 live births) [7]. Omphaloceles also have a higher prevalence in male newborns and are associated with multiple births [6]. The risk associated with in utero exposure to selective serotonin reuptake inhibitor (SSRI) of omphalocele has been controversial [8, 9].

We present the case of a 37-year-old pregnant woman with an early diagnosis of liver-containing omphalocele associating ductus venosus reversed flow, with the aim to highlight the importance of the first-trimester morphology scan and to develop a pilot study regarding the neurological development of infants after surgical repair of giant omphaloceles.

## Case Report

**Figure 1.**
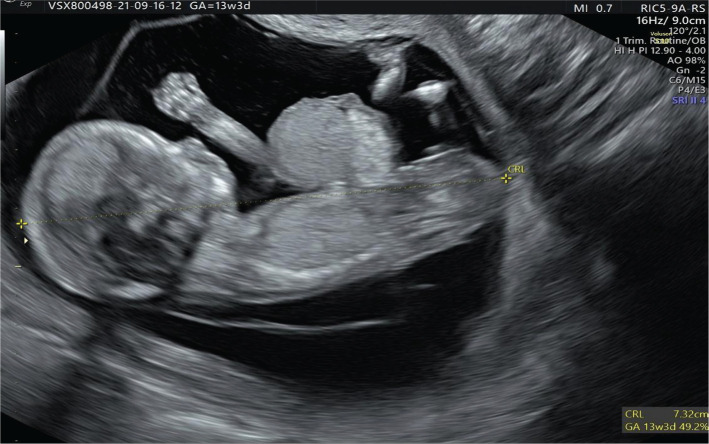
Crown-rump length (CRL).

**Figure 2.**
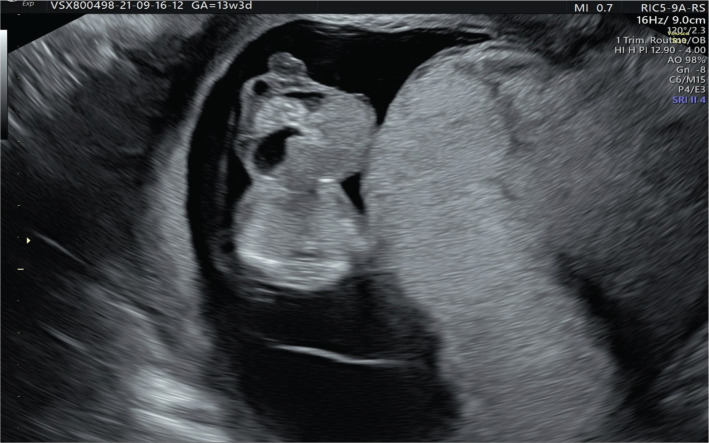
Omphalocele containing bowel, liver and stomach.

**Figure 3.**
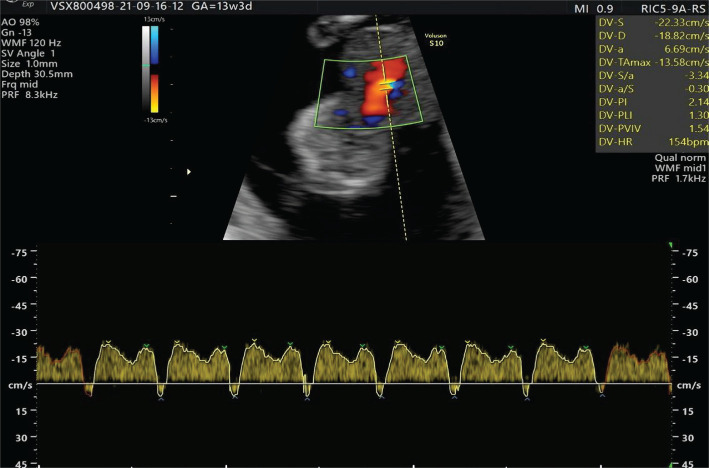
Ductus venosus reversed flow.

A 37-year-old woman presented to our medical unit for early diagnosis of pregnancy and pregnancy monitoring. We noted nothing abnormal from the physical, ultrasonographic examination, and the patient’s medical and obstetrical history. At 10 weeks of gestation, the patient returned for an ultrasonographic examination in which a physiologic hernia was observed. During the fetal morphology scan at 13 weeks and 3 days of gestation, a 1,02 cm abdominal wall defect was detected: a liver-containing omphalocele (Figure 1 and 2). The ductus venosus flow was evaluated during the morphology scan, and the fetus presented ductus venosus reversed flow (Figure 3). No other morphological abnormalities were detected when transvaginal ultrasonography was performed. The patient was recommended to undergo a non-invasive prenatal screening test, but she refused. The patient was informed about the risks of neonatal complications, and the reconstruction possibilities implied, and she decided to terminate the pregnancy. A karyotype evaluation of the conception product was performed, and the result was negative for chromosomal abnormalities.

## Discussion

Over 90% of cases of omphalocele are diagnosed prenatally during a standard obstetric ultrasonographic examination [10]. Usually, non-liver-containing omphalocele can be properly diagnosed after 12 weeks of gestation, thus diagnosing correctly the cases of a small omphalocele, which can be easily mistaken for physiologic midgut herniation. On the other hand, liver-containing omphaloceles can be diagnosed earlier using transvaginal ultrasonography at 9 or 10 weeks of gestation due to the well-known fact that the liver does not herniate physiologically [11, 12]. The ultrasonographic findings include a midline defect of the abdominal wall in the umbilical area, of varying size, a membranous sac usually containing bowel, livers, stomach, or even bladder [12]. The sac comprises the amnion representing the exterior layer and Wharton’s jelly in the middle and peritoneum – the interior layer. The fetus might present ascites in the abdominal cavity or the sac. The sac could contain ductus venosus that can be evaluated using color Doppler [11]. Small omphaloceles, non-liver-containing, are usually correlated with fetal aneuploidies, while omphaloceles with the liver in the sac are commonly affiliated with euploid fetuses [13]. The most frequent pathologies we should consider while making a differential diagnosis are represented by: gastroschisis, umbilical cord hernia, ectopia cordis, cloacal exstrophy, limb-body wall complex, Pentalogy of Cantrell or urachal cysts [10].

Omphaloceles are in a proportion of 35% to 70% associated with structural anomalies such as tetralogy of Fallot, dextrocardia, malrotation, intestinal atresia, ventricular septal defect, bladder or renal agenesis, ureteral stenosis orofacial clefts, diaphragmatic and neural tube defects [14–16]. 60% of non-liver-containing omphaloceles are linked to fetal aneuploidies such as trisomy 13, 18, or 21, triploids, Turner syndrome, and rare chromosomal deletions [17, 18]. One-third of pregnancies with omphaloceles present polyhydramnios after the 20^th^ gestational week; fetal growth restriction is also associated with omphalocele [17, 19]. Regarding syndromes, omphalocele is encountered in Beckwith-Wiedemann syndrome, Schisis association, Pentalogy of Cantrell, OEIS (omphalocele, exstrophy of the bladder, imperforate anus, spinal defects) complex, Donnai-Barrow syndrome, and Shprintzen-Goldberg omphalocele syndrome [20].

After a positive diagnosis of omphalocele, complementary investigations should be recommended: microarray, given the high risk of aneuploidy, fetal echocardiogram due to elevated incidence of associated cardiac abnormalities, and testing for Beckwith-Wiedemann syndrome due to its increased risk in euploid fetuses presenting omphalocele, but only after a negative microarray test for aneuploidies [21].

The next step after the positive diagnosis of omphalocele is to counsel and inform the parents about the implications of omphaloceles associated or not with congenital syndromes or congenital abnormalities. Also, the parents might choose to terminate the pregnancy, and a pathology report will be realized to establish the omphalocele’s etiology and avoid recurrence in future pregnancies. Cases implying omphalocele associated with trisomy 13, 18, triploidy, or pulmonary hypoplasia from Pentalogy of Cantrell have an obscure prognosis [22–24].

The pregnancy monitoring consists of ultrasonographic examinations to follow the fetal growth every three to four weeks and nonstress test or biophysical profile monitoring starting with the 32^nd^ week of gestation in the cases with normal growth and adequate amniotic fluid index (AFI). Intrauterine growth restriction appears frequent in pregnancies with omphalocele and associated abnormalities, presenting an elevated risk of unfortunate neonatal outcomes [25]. The standard biometry measurements include abdominal circumference (AC), biparietal diameter, head circumference, and femur length. In case of inconsistent or diminished AC, the other parameters (femur length, biparietal diameter) and AFI should be closely evaluated. A specific formula to assess the fetal weight in case of abdominal wall defects was elaborated by Siemer *et al.* [26], which estimates the fetal weight using the occipitofrontal diameter, biparietal diameter, and femur length measurements. Regarding the location, the newborn should be delivered at a tertiary care center, vaginal delivery should be attempted, in the absence of standard indications for C-section; cesarean delivery is recommended in cases of fetuses with giant omphaloceles [27–29].

When the newborn is delivered, it is essential to avoid clamping the umbilical sac to prevent accidents of bowel injury. Further, the newborn with omphalocele requires immediate care in order to be placed in a thermoneutral environment. The surgery is discussed: for small abdominal wall defects measuring 2 to 3 cm, the surgery occurs in the first 24–72 hours of life and consists of primary closure of the fascia and skin [30]. Larger defects usually benefit from silo placing over the defect in the first 24 hours of life, and the closure is delayed. After three to seven days, the silo is reduced progressively in the intensive care unit, and the definite closure of the abdominal wall defect occurs in the operating room [30]. Reducing liver-containing omphaloceles should be done under Doppler ultrasound guidance to ensure that silo reduction does not damage the vena cava and hepatic outflow [30].

Regarding giant omphaloceles, the management could include acellular dermal patch, silo, and skin graft or sclerosing solution (topical povidone-iodine) application, which aids in amniotic sacs eschar formation, followed by delayed hernia repair [30]. The infant might need prolonged mechanical ventilation to manage compression of the vena cava and respiratory difficulties due to omphalocele reduction. Moreover, postoperative surveillance must include urine output, blood pressure, and pulse rate monitoring [31]. Multiple reconstructive procedures may be mandatory in the cases of a newborn with large liver-containing omphaloceles, which could have a role in the long-term morbidity [31].

Regarding the consequences of ductus venosus reversed velocity, Caradeaux *et al.* [32] reported an 11.6 odds ratio for fetal death for fetuses with absent ductus venosus or reversed end-diastolic velocity. Alves *et al.* [33] associated fetuses with absent or ductus venosus reversed flow with the following postnatal outcomes: low birth weight, lower Apgar scores at 1 and 5 minutes. These newborns with absent or ductus venosus reversed flow present a higher incidence of orotracheal intubation, pH at birth less than 7.2, pulmonary hemorrhage, thrombocytopenia, intracranial hemorrhage, hypoglycemia, and postnatal death.

Besides being a marker of fetal cardiac malformations, many authors [34–36] have associated ductus venosus reversed flow with fetal hypoxia in cases of a poorer perinatal outcome as well as intrauterine growth restriction. The TRUFFLE study by Visser *et al.* [37] concluded that fetuses delivered prematurely based on the first appearance of an abnormal ductus venosus waveform have a better neurological development at the 2-year follow-up than those delivered due to an abnormal cardiotocography. Therefore, we can hypothesize that the ductus venosus reversed flow is an early marker of fetal hypoxia.

## Conclusion

The particularity of this case consists of a fetus with a positive diagnosis of a giant liver-containing omphalocele but with a small abdominal wall defect during the first-trimester morphology scan at 13 weeks and 3 days of gestation which associated ductus venosus reversed flow, presenting a normal karyotype post abortum. With a small defect, we can speculate the risk of strangling besides the mechanical traction exercised on the ductus venosus generating fetal distress, specifically fetal hypoxia at an early gestational age.

In conclusion, the main issue, in this case, is if the fetal omphalocele and ductus venosus reversed flow indicate fetal hypoxia, what is the obstruction effect on the oxygenated blood pathway caused by the abdominal defect, and which are the long-term effects on infants with this complex pathology with an unknown outcome.

## Acknowledgments

### Conflict of interest

The authors declare that there is no conflict of interest.

### Consent for publication

Informed consent to publish the data was obtained from the participant in this case report.

### Authorship

All the authors contributed equally to this work.

## References

[1] Duhamel B (1963). Embryology of Exomphalos and Allied Malformations. Arch Dis Child.

[2] Duke DS, Puri P. HM (2009). SMZ. Omphalocele and Gastroschisis. Pediatric Surgery.

[3] Verla MA, Style CC, Olutoye OO (2019). Prenatal diagnosis and management of omphalocele. Semin Pediatr Surg.

[4] Stallings EB, Isenburg JL, Short TD, Heinke D, Kirby RS, Romitti PA, Canfield MA, O’Leary LA, Liberman RF, Forestieri NE, Nembhard WN, Sandidge T, Nestoridi E, Salemi JL, Nance AE, Duckett K, Ramirez GM, Shan X, Shi J, Lupo PJ (2019). Population-based birth defects data in the United States, 2012-2016: A focus on abdominal wall defects. Birth Defects Res.

[5] Byron-Scott R, Haan E, Chan A, Bower C, Scott H, Clark K (1998). A population-based study of abdominal wall defects in South Australia and Western Australia. Paediatr Perinat Epidemiol.

[6] Marshall J, Salemi JL, Tanner JP, Ramakrishnan R, Feldkamp ML, Marengo LK, Meyer RE, Druschel CM, Rickard R, Kirby RS, National Birth Defects Prevention Network. (2015). Prevalence, Correlates, and Outcomes of Omphalocele in the United States, 1995-2005. Obstet Gynecol.

[7] Kirby RS (2017). The prevalence of selected major birth defects in the United States. Semin Perinatol.

[8] Waller DK, Shaw GM, Rasmussen SA, Hobbs CA, Canfield MA, Siega-Riz AM, Gallaway MS, Correa A, National Birth Defects Prevention Study (2007). Prepregnancy obesity as a risk factor for structural birth defects. Arch Pediatr Adolesc Med.

[9] Alwan S, Reefhuis J, Rasmussen SA, Olney RS, Friedman JM, National Birth Defects Prevention Study (2007). Use of selective serotonin-reuptake inhibitors in pregnancy and the risk of birth defects. N Engl J Med.

[10] Romero R, Romero R (1998). Omphalocele. Prenatal diagnosis of congenital anomalies.

[11] Curtis JA, Watson L (1988). Sonographic diagnosis of omphalocele in the first trimester of fetal gestation. J Ultrasound Med.

[12] Brown DL, Emerson DS, Shulman LP, Carson SA (1989). Sonographic diagnosis of omphalocele during 10^th^ week of gestation. AJR Am J Roentgenol.

[13] Nyberg DA, Fitzsimmons J, Mack LA, Hughes M, Pretorius DH, Hickok D, Shepard TH (1989). Chromosomal abnormalities in fetuses with omphalocele. Significance of omphalocele contents. J Ultrasound Med.

[14] Ardinger HH, Williamson RA, Grant S (1987). Association of neural tube defects with omphalocele in chromosomally normal fetuses. Am J Med Genet.

[15] Fogel M, Copel JA, Cullen MT, Hobbins JC, Kleinman CS (1991). Congenital heart disease and fetal thoracoabdominal anomalies: associations in utero and the importance of cytogenetic analysis. Am J Perinatol.

[16] Henrich K, Huemmer HP, Reingruber B, Weber PG (2008). Gastroschisis and omphalocele: treatments and long-term outcomes. Pediatr Surg Int.

[17] Lakasing L, Cicero S, Davenport M, Patel S, Nicolaides KH (2006). Current outcome of antenatally diagnosed exomphalos: an 11 year review. J Pediatr Surg.

[18] van Zalen-Sprock RM, Vugt JM, van Geijn HP (1997). First-trimester sonography of physiological midgut herniation and early diagnosis of omphalocele. Prenat Diagn.

[19] Ozawa K, Ishikawa H, Maruyama Y, Nagata T, Nagase H, Itani Y, Kurosawa K, Yamanaka M (2011). Congenital omphalocele and polyhydramnios: a study of 52 cases. Fetal Diagn Ther.

[20] Chen CP (2007). Syndromes and disorders associated with omphalocele (III): single gene disorders, neural tube defects, diaphragmatic defects and others. Taiwan J Obstet Gynecol.

[21] Porter A, Benson CB, Hawley P, Wilkins-Haug L (2009). Outcome of fetuses with a prenatal ultrasound diagnosis of isolated omphalocele. Prenat Diagn.

[22] Brantberg A, Blaas HG, Haugen SE, Eik-Nes SH (2005). Characteristics and outcome of 90 cases of fetal omphalocele. Ultrasound Obstet Gynecol.

[23] Heider AL, Strauss RA, Kuller JA (2004). Omphalocele: clinical outcomes in cases with normal karyotypes. Am J Obstet Gynecol.

[24] Poznanski AK (1990). Fetal omphalocele: prenatal US detection of concurrent anomalies and other predictors of outcome. Radiology.

[25] Nicholas SS, Stamilio DM, Dicke JM, Gray DL, Macones GA, Odibo AO (2009). Predicting adverse neonatal outcomes in fetuses with abdominal wall defects using prenatal risk factors. Am J Obstet Gynecol.

[26] Siemer J, Hilbert A, Hart N, Hoopmann M, Schneider U, Girschick G, Müller A, Schild RL (2008). Specific weight formula for fetuses with abdominal wall defects. Ultrasound Obstet Gynecol.

[27] Carpenter MW, Curci MR, Dibbins AW, Haddow JE (1984). Perinatal management of ventral wall defects. Obstet Gynecol.

[28] How HY, Harris BJ, Pietrantoni M, Evans JC, Dutton S, Khoury J, Siddiqi TA (2000). Is vaginal delivery preferable to elective cesarean delivery in fetuses with a known ventral wall defect?. Am J Obstet Gynecol.

[29] Segel SY, Marder SJ, Parry S, Macones GA (2001). Fetal abdominal wall defects and mode of delivery: a systematic review. Obstet Gynecol.

[30] Pacilli M, Spitz L, Kiely EM, Curry J, Pierro A (2005). Staged repair of giant omphalocele in the neonatal period. J Pediatr Surg.

[31] Biard JM, Wilson RD, Johnson MP, Hedrick HL, Schwarz U, Flake AW, Crombleholme TM, Adzick NS (2004). Prenatally diagnosed giant omphaloceles: short- and long-term outcomes. Prenat Diagn.

[32] Caradeux J, Martinez-Portilla RJ, Basuki TR, Kiserud T, Figueras F (2018). Risk of fetal death in growth-restricted fetuses with umbilical and/or ductus venosus absent or reversed end-diastolic velocities before 34 weeks of gestation: a systematic review and meta-analysis. Am J Obstet Gynecol.

[33] Alves SK, Francisco RP, Miyadahira S, Krebs VL, Vaz FA, Zugaib M (2008). Ductus venosus Doppler and postnatal outcomes in fetuses with absent or reversed end-diastolic flow in the umbilical arteries. Eur J Obstet Gynecol Reprod Biol.

[34] Ferrazzi E, Lees C, Acharya G (2019). The controversial role of the ductus venosus in hypoxic human fetuses. Acta Obstet Gynecol Scand.

[35] Rizzo G, Capponi A, Pietrolucci ME, Boccia C, Arduini D (2009). The significance of visualising coronary blood flow in early onset severe growth restricted fetuses with reverse flow in the ductus venosus. The Journal of Maternal-Fetal & Neonatal Medicine.

[36] Müller T, Nanan R, Rehn M, Kristen P, Dietl J (2002). Arterial and ductus venosus Doppler in fetuses with absent or reverse end-diastolic flow in the umbilical artery: correlation with short-term perinatal outcome. Acta obstetricia et gynecologica Scandinavica.

[37] Visser GHA, Bilardo CM, Derks JB, Ferrazzi E, Fratelli N, Frusca T, Ganzevoort W, Lees CC, Napolitano R, Todros T, Wolf H, Hecher K, TRUFFLE group investigators (2017). Fetal monitoring indications for delivery and 2-year outcome in 310 infants with fetal growth restriction delivered before 32 weeks’ gestation in the TRUFFLE study. Ultrasound Obstet Gynecol.

